# Association of loneliness and social isolation with all-cause mortality among older Mexican adults in the Mexican health and aging study: a retrospective observational study

**DOI:** 10.1186/s12877-023-03750-3

**Published:** 2023-01-25

**Authors:** Ashuin Kammar-García, Ricardo Ramírez-Aldana, Paloma Roa-Rojas, Luis Raymundo Lozano-Juárez, Sergio Sánchez-García, Pamela Tella-Vega, Carmen García-Peña

**Affiliations:** 1 Health Research Division, Instituto Nacional de Geriatría, Mexico City, Mexico; 2 Public Policy Laboratory, Instituto Nacional de Geriatría, Mexico City, Mexico; 3 Epidemiological Research Unit and Health Services, Aging Area, National Medical Center XXI Century, Instituto Mexicano del Seguro Social, Mexico City, Mexico; 4 Health Research Director, Instituto Nacional de Geriatría, Mexico City, Mexico

**Keywords:** Loneliness, Social isolation, Mortality, Older adults, Mental health

## Abstract

**Background:**

Plenty of evidence shows how social isolation and loneliness are associated with increased risk for numerous diseases and mortality. But findings about their interactive or combined effects on health outcomes and mortality remains inconclusive.

**Objective:**

Analyze the longitudinal association of loneliness, social isolation and their interactions, with the all-cause mortality among older adults in Mexico.

**Methods:**

A retrospective observational study was conducted. Mexican adults older than 50 years were included. Data from the Mexican Health and Aging Study (MHAS) in the 2015 and 2018 waves were used. The subjects were classified according to their level of loneliness and the presence of social isolation. Multivariate logistic regression analyzes were performed to determine the degree of association between loneliness and social isolation with all-cause mortality at a 3-year follow-up.

**Results:**

From the total sample of 11,713 adults aged 50 years or over, 707 (6%) did not survive, 42% presented loneliness, and 53% were classified as socially isolated. After multivariate adjustment only social isolation (OR = 1.30, 95%CI:1.03–1.64) was associated with all-cause mortality, loneliness (Mild: OR = 0.83, 95%CI:0.59–1.16; Severe: OR = 1.03, 95%CI:0.71–1.64), and the interaction between loneliness and social isolation were not associated with all-cause mortality.

**Conclusion:**

Social isolation, but not loneliness or their interaction, was associated with all-cause mortality in Mexican adults older than 50 years. This finding may help direct possible future interventions that help improve mental health in older adults from a highly collectivistic country.

## Background

Older persons are the fastest growing segment of the population worldwide. The World Health Organization (WHO) calculates that by 2050 one in every five people throughout the world will be 60 years of age or older. Among these, 80% will be living in low to middle income countries [1]. This demographic transition poses a burden to the individual and to his or her family, as well as to the society and represents a challenge for public health systems, especially in countries such as México [2], where 7.8% of its population is older than 65 years [3].

Loneliness has been defined as a feeling of isolation despite having a social network present [4] and can be considered as a subjective characteristic of the satisfaction that an individual feels about the quality of social relationships [5], while social isolation is defined as the presence of low quantity and quality of contact with other people [4] and is an objective characteristic on the count of social contacts of an individual [5]. Although social isolation could be related to loneliness [6], a low correlation and discrepancies between loneliness and social isolation have recently been observed [7].

Increasing age is considered a risk factor for loneliness and social isolation [4, 6]. The prevalence of loneliness in older adults is estimated between 10 and 50% [4], and chronic loneliness prevalence have been reported in 15–30% of older adults and occasional loneliness up to 60–80% [8]. And regarding social isolation of older adults, the prevalence is estimated at 6–43% [4]. In Mexico, the prevalence of loneliness is estimated at 13.2–34.9% [9, 10], and of social isolation at 34–43% [6, 11], both in older adults.

Plenty of evidence shows that social isolation and loneliness are associated with increased risk for numerous diseases, such as: cardio metabolic diseases in general population [12, 13], infectious diseases in patients aged 18 to 55 years [14], sleep alterations in general population [15], cognitive decline in older adults [16] and depression in older adults [17, 18]. Moreover, isolation and loneliness negatively impact the self-perception of health [19].

Conflicting results have been obtained through different studies for the role of loneliness or social isolation and mortality, some studies show an increased risk of mortality in older adults [20, 21], while others do not conclude that loneliness or social isolation are determining factors of fatal outcomes in the same population [22–24]. Although social isolation and loneliness often coexist, findings about their interactive or combined effects on health outcomes and mortality remains inconclusive [25]. Some studies have found a combined or synergic effect between social isolation and loneliness in middle-age and older adults [26, 27], other studies have found that the combination of loneliness and social isolation predict all-cause mortality in older adults [5], but the combined effect is not worse than experiencing either by itself [28, 29]. It remains unclear how these two aspects of human socialization interact with each other.

Currently, most previous studies come from high-income [20, 21, 24, 30], highly individualistic countries [31]. Given that there are cultural and socioeconomic factors that influence the effect of loneliness and social isolation on health and all-cause mortality risk [32], it is necessary to research highly collectivistic middle- and low-income countries, like Mexico, which can be used as a baseline for the study of the consequences of this phenomenon on wider spectrum of countries in Latin-American that share a similar family centered culture. Therefore, the aim for this study is to analyze the longitudinal association of loneliness, social isolation, and their interactions, with the all-cause mortality among older adults in Mexico.

## Methods

### Study design

An observational retrospective cohort study was carried out in which Mexican adults older than 50 years were included. The exclusion criteria were the lack of information on rates of loneliness and social isolation. Subjects with incomplete information were excluded from the study.

### Source of data

Data from the Mexican Health and Aging Study (MHAS) were used, which is a prospective cohort study initiated in 2001 with the aim of examining the aging process and its disease and disability burden; the study methodology has already been described previously [33, 34]. The MHAS has had several waves of data collection and evaluation as well as updating of the sample, in this study the data from the evaluation carried out in 2015 were used to obtain information on loneliness, social isolation and the rest of the sociodemographic, clinical, psychological and lifestyle characteristics. All-cause mortality information was obtained from the evaluation carried out in 2018. The MHAS was approved by the Institutional Review Boards and Ethics Committees of the University of Texas Medical Branch in the USA, the National Institute of Statistics and Geography (INEGI), and the National Institute of Public Health (INSP) in Mexico. The current study was approved by the National Institute of Geriatrics (DI-PI-007/2021).

### Exposures

The exposure variables in this study were loneliness and social isolation, information from the 2015 wave was used.

Loneliness was assessed using the Revised UCLA Loneliness Scale (UCLA-LS) [35], which has been widely used in other studies [16, 27, 36], and its validity has also been evaluated elsewhere [37]. This scale has three items: 1) How often do you need company? 2) How often do you feel left out? 3) How often do you feel isolated? Each of the items admits answers from 1 “almost never” to 3 “often”. The total score of the three items results in an index with values from 3 to 9. Three categories are made: a score of 3 represents no loneliness (without); 4 to 5 mild loneliness; and, 6 to 9 severe loneliness, where 9, logically, indicates the most loneliness [38].

Social isolation was assessed by Berkman and Syme’s Social Network Index (SNI) [39]. The SNI is composed by four dichotomic items: housing, religious activity, groups, and closeness. 1) Housing: this item assigns a value of 1 to individuals with a couple and 0 to individuals without a couple. 2) Religious Activity: attending church at least once a month is given a value of 1, attending less than once a month or occasional attendance, receives a value of 0. 3) Groups: being an active member of any group, including religious ones, is given a value of 1, not being an active member of any group is given a value of 0. 4) Closeness: it assesses the number of relatives, kin, or close friends the person has. It produces a value from zero to three, where a value of 1 is given for being close with others and a value of 0 is given for not being close with others. The sum of the scores for these four individual items results in a final index. We used a reverse code to emphasize those who had higher scores as more isolated, where 0 represents a larger social network and 4 the absence of it. A value from 2 to 4 corresponds to an isolated individual, whereas a value of 0 or 1 speaks of an integrated individual. Cut-off points for evaluating social isolation were taken from Domènech-Abella J, 2019 [40].

### Sociodemographic, clinical, psychological and lifestyle characteristics

For all Sociodemographic, clinical, psychological and lifestyle characteristics, the information from the 2015 wave was used.

The sociodemographic variables included in the study were age, weight and height (self reported), BMI, sex, socioeconomic level, years of schooling and the self-reported living alone status, for the living alone status, two categories were made. 1) Living alone: those without a couple or without anyone else living in the same household, and 2) Not living alone: those with a couple or other people living in the same household.

The self-reported clinical characteristics included in the study were comorbidities previously diagnosed by medical physicians (diabetes, hypertension, heart attack, lung chronic disease [asthma or emphysema], stroke and infectious diseases [including kidney infection, liver infection, tuberculosis, pneumonia and herpes or herpes zoster]), self-reported of activities of daily living affected or that need assistance (walking, bathing, eating, use of toilet, and getting into or out of bed) evaluated by the basic activities of daily living (ADL) questionnaire (categorized as one or more ADLs affected), [41]; self-reported of falls (defined as one or more falls in the las two years), hearing problems (defined as poor hearing or legally deaf), sight problems (defined as a poor sigh or legally blind), presence of limiting pain (defined as pain that limits the participant’s usual activities), unintentional weight loss (defined as unwanted weight loss of 5 Kg or more in the last two years), hospitalization (defined as frequency of hospitalizations in the previous year categorized as none hospitalization, one to five hospitalization and more than five hospitalizations.).

The psychological characteristics included in this study were depressive symptoms, cognition, emotional locus of control, and life satisfaction. Prescence depressive symptoms were assessed with the 9-item version of the Center of Epidemiological studies-Depression questionnaire (CESD-9) [42]. The cut- off point, validated for Mexican population, positive to depressive symptoms was a score of 5 or higher [43]. Cognition was measured with the Mexican version of the Cross-Cultural Cognitive Examination (CCCE) [44]. Emotional locus of control, defined as the individual’s beliefs regarding the extent which he or she is able to control or influence important life events including health outcomes [45], was measured with an adapted measure of Rotter’s scale [46], with values range from 4 to 16, where the higher the score the more he or she has a sense that he or she is in control of his or her own life. Satisfaction with life was measured using the response to the statement “I am satisfied with my life”, where she or he could agree, disagree, or remain neutral.

Lifestyle characteristics included were smoking and alcohol use. We classified the participants in three categories according to their smoking habits (has never smoked, former smoker, and current smoker) [47]; and according to daily alcohol intake considering the sex into: “Has never used” for people that have never consumed alcohol, “Currently doesn’t use or mild use”, “Moderate user” (1 alcoholic beverage for women per day and, 1 or 2 for men per day), and “Heavy user” (2 or more alcoholic beverage for women per day and, 3 or more for men per day) [48].

### All-cause mortality

A face-to-face interview was conducted in all the homes of the participants during the year 2018, the interview was carried out with the relatives of the participant, and all-cause mortality during the 3-year follow-up period were registered, no loss to follow-up was recorded in the participants.

### Statistical analysis

Descriptive data is presented as mean and standard deviation (SD) together with its standardized mean (Z score) for quantitative data, and as frequency and percentage for qualitative data. Comparisons of quantitative variables between groups of exposition were made by t-test and one-way Welch’s ANOVA, poshoc comparison were made by the Dunnett’s t test. Comparison of qualitative variables between groups of exposition were made by chi-square test. Comparison of all-cause mortality incidences between levels of loneliness and between socially integrated and socially isolated subjects were made by the chi-square test. Kendall, gamma and Spearman correlation analyzes were applied to determine the correlation between loneliness and social isolation scores, similarly, Cramer’s test was applied to determine the degree of association between the categories of loneliness and social isolation, and the variable living alone with loneliness and social isolation.

Univariate logistic regression analyzes were applied to determine the association of various social, clinical, and psychological characteristics with all-cause mortality; variables associated with all-cause mortality with a *p* value ≤0.05 were included for adjustment of a logistic regression model in which included the levels of loneliness, social isolation, and their interactions as all-cause mortality predictors. The model was adjusted for coviables that have previously shown an association with mortality in older adults: age, sex, schooling, more than one activity of daily living affected, presence of depressive symptoms, satisfaction with life, internal locus of control, multimorbidity (defined as 2 or more comorbidities present in the same participant), infectious diseases, falls, sight problems, hearing problems, limiting pain, smoking, alcohol consumption, unintended weight loss, hospitalization and living alone. A sensitivity analyses were conducted considering the loneliness as a binary variable (Without loneliness vs Mild and severe loneliness).

The assumptions of the multivariates models were verified by collinearity tests, residual analysis, and calculation of the goodness of fit. The results are summarized as Odds Ratio (OR) and 95% confidence intervals (95%CI).

A value of *p* < 0.05 was considered as statistical significance. No data imputations were performed. All analyzes were performed with Stata and SPSS v.21 statistical software.

## Results

**Fig. 1:**
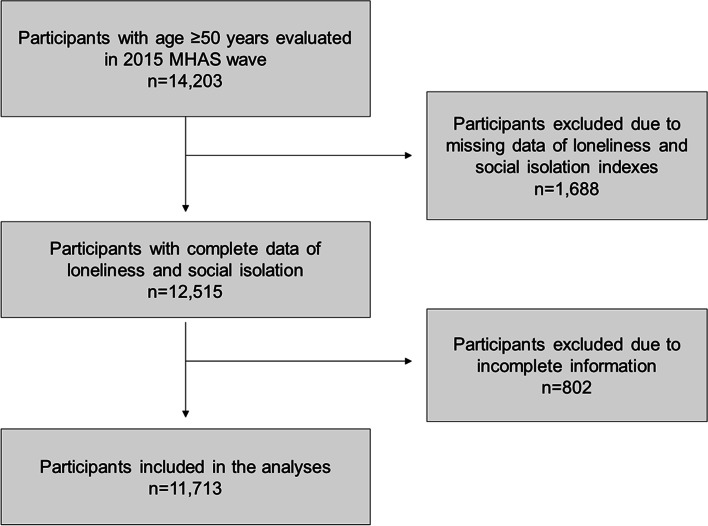
Flow diagram of participants assessed for eligibility

A total of 14,203 subjects were included in the study, of which 2490 were excluded. The final sample was 11,713 subjects. Figure 1 shows the flow chart for obtaining the final sample. The mean age was 66.6 (SD: 9.37) years. 58.2% (*n* = 6817) of the subjects were women. 86.7% of subjects had no prior hospitalizations, 9.5% of subjects had 1 to 5 prior hospitalizations, and only 3.9% of subjects had more than 5 prior hospitalizations. The incidence of all-cause mortality in the 3 years of follow-up was 6.0% (95%CI: 5.6–6.5) (*n* = 707).

The mean loneliness score was 4.03 (SD: 1.54, Z score: − 0.0035, Z score SD: 0.80), and the social isolation score was 1.65 (SD: 0.80, Z score: − 0.01, Z score SD: 0.99). Of the 11,713 participants, 42.05% presented some degree of loneliness, and 53.34% were classified as socially isolated.

Table 1 shows the sociodemographic, clinical, psychological and lifestyle characteristics in the total sample and according to the classification of loneliness and social isolation. As the level of loneliness increases, the financial situation is worse, similar in the presence of depressive symptoms, and satisfaction with life. The frequency of falls is also higher in subjects with some degree of loneliness. The situation is similar with participants with social isolation, a higher proportion of a poor financial situation is observed in isolated subjects, but not in the rest of the categories. The presence of depressive symptoms is higher in subjects with social isolation, but satisfaction with life is similar, as is the frequency of falls. The presence of multimorbidity increases with the level of loneliness, but it is not higher among subjects with social isolation. Unintentional weight loss is greater in subjects with greater loneliness, but only 3% greater in subjects with isolation than those who are socially integrated. Of the 5466 subjects with social isolation, only 17% live alone, like the 14 and 16% of subjects with mild or severe loneliness who live alone.

**Table 1:** Sociodemographic, clinical, psychological and lifestyle characteristics according to degrees of loneliness and social isolation

	Loneliness					Social isolation		
	Total sample ***n*** = 11,713	Without ***n*** = 6788	Mild ***n*** = 2964	Severe ***n*** = 1961	***p*** Value	Isolated ***n*** = 5466	Integrated ***n*** = 6247	***p*** Value
**Age, years**	66.6 (9.34)	65.85 (8.9)	67.17 (9.62)^a^	68.44 (9.86)^a^	< 0.0001	68.10 (9.82)	64.92 (8.44)	< 0.0001
**Age categories, n (%)**								
50–59	3113 (26.58)	1941 (28.6)	751 (25.3)	421 (21.5)	< 0.0001	1440 (23.1)	1673 (30.6)	< 0.0001
60–69	4335 (37.01)	2601 (38.3)	1054 (35.6)	680 (34.7)		2117 (33.9)	2218 (40.6)	
70–79	3092 (26.40)	1694 (25.0)	831 (28.0)	567 (28.9)		1824 (29.2)	1268 (23.2)	
≥ 80	1173 (10.01)	552 (8.1)	328 (11.1)	293 (14.9)		866 (13.9)	307 (5.6)	
**Height, m**	1.59 (0.09)	1.60 (0.09)	1.58 (0.10)^a^	1.57 (0.09)^a^	< 0.0001	1.58 (0.10)	1.60 (0.09)	< 0.0001
**Weight, Kg**	69.84 (14.08)	70.7 (14.0)	69.1 (13.8)^a^	67.8 (14.5)^a^	< 0.0001	68.37 (14.37)	71.5 (13.6)	< 0.0001
**Body mass index**	27.5 (4.87)	27.5 (4.68)	27.5 (5.06)	27.5 (5.3)	0.95	27.28 (5.00)	27.8 (4.72)	< 0.0001
**Sex, n (%)**								
Men	4896 (41.80)	3134 (46.2)	1131 (38.2)	631 (32.2)	< 0.0001	2319 (37.1)	2577 (47.1)	< 0.0001
Women	6817 (58.20)	3654 (53.8)	1833 (61.8)	1330 (67.8)		3928 (62.9)	2889 (52.9)	
**Schooling, years**	5.76 (4.68)	6.42 (4.89)	5.34 (4.38)^a^	4.13 (3.82)^a^	< 0.0001	5.40 (4.55)	6.18 (4.78)	< 0.0001
**Schooling, n (%)**								
No schooling	3681 (31.43)	2503 (36.9)	834 (28.1)	344 (17.5)	< 0.0001	1822 (29.2)	1859 (34.0)	< 0.0001
1–6 years	6149 (52.50)	3382 (49.8)	1635 (55.2)	1132 (57.7)		3285 (52.6)	2864 (52.4)	
≥ 7 years	1883 (16.08)	903 (13.3)	495(16.7)	485 (24.7)		1140 (18.2)	743 (13.6)	
**Financial situation, n (%)**								
Excellent	110 (0.94)	76 (1.1)	20 (0.7)	14 (0.7)	< 0.0001	53 (0.8)	57 (1.0)	< 0.0001
Very Good	155 (1.32)	116 (1.7)	31 (1.0)	8 (0.4)		73 (1.2)	82 (1.5)	
Good	2528 (21.58)	1733 (25.5)	561 (18.9)	234 (11.9)		1287 (20.6)	1241 (22.7)	
Fair	7627 (65.12)	4359 (64.2)	1999 (67.4)	1269 (64.7)		4024 (64.4)	3603 (65.9)	
Poor	1293 (11.04)	504 (7.4)	353 (11.9)	436 (22.2)		810 (13.0)	483 (8.8)	
**Activities of Daily Living affected, n (%)**								
None activity affected	11,191 (95.5)	6603 (97.3)	2809 (94.8)	1779 (90.7)	< 0.0001	5275 (96.5)	5916 (94.7)	< 0.0001
More than 1 activity affected	522 (4.5)	185 (2.7)	155 (5.2)	182 (9.3)		191 (3.5)	331 (5.3)	
**Type of activities affected, n (%)**								
Walking	197 (1.68)	70 (1.0)	57 (1.9)	70 (3.6)	< 0.0001	134 (2.1)	63 (1.2)	< 0.0001
Bathing	291 (2.48)	101 (1.5)	94 (3.2)	96 (4.9)	< 0.0001	196 (3.1)	95 (1.7)	< 0.0001
Eating	173 (1.5)	53 (0.8)	57 (1.9)	63 (3.2)	< 0.0001	113 (1.8)	60 (1.1)	0.001
Using the toilet	174 (1.5)	60 (0.9)	60 (2.0)	54 (2.8)	< 0.0001	116 (1.9)	58 (1.1)	< 0.0001
Getting into or out of bed	247 (2.1)	80 (1.2)	83 (2.8)	84 (4.3)	< 0.0001	151 (2.4)	96 (1.8)	0.01
**Cognition, score**	0.004 (0.99)	0.13 (0.97)	−0.10 (0.96)^a^	− 0.31 (0.96)^a^	< 0.0001	− 0.10 (1.00)	0.12 (0.94)	< 0.0001
**Depressive symptoms, n (%)**	3615 (30.86)	1012 (14.9)	1200 (40.5)	1403 (71.5)	< 0.0001	2157 (34.5)	1458 (26.7)	< 0.0001
**Life Satisfaction, n (%)**								
Agrees	10,057 (85.86)	6206 (91.4)	2479 (83.6)	1372 (70.0)	< 0.0001	5224 (83.6)	4833 (88.4)	< 0.0001
Remains neutral	978 (8.35)	385 (5.7)	307 (10.4)	286 (14.6)		588 (9.4)	390 (7.1)	
Disagrees	678 (5.79)	197 (2.9)	178 (6.0)	303 (15.5)		435 (7.0)	243 (4.4)	
**Internal locus of control, score**	5.34 (1.78)	5.25 (1.86)	5.38 (1.78)^a^	5.58 (2.04)^a^	< 0.0001	5.42 (1.84)	5.25 (1.69)	< 0.0001
**Comorbidities, n (%)**								
Hypertension	5735 (48.96)	3107 (45.8)	1527 (51.5)	1101 (56.1)	< 0.0001	3124 (50.0)	2611 (47.8)	0.02
Diabetes	3003 (25.64)	1598 (23.5)	799 (27.0)	606 (30.9)	< 0.0001	1608 (25.7)	1395 (25.5)	0.79
Cancer	284 (2.42)	158 (2.3)	63 (2.1)	63 (3.2)	0.08	129 (2.1)	155 (2.8)	0.007
Respiratory disease	730 (6.23)	341 (5.0)	219 (7.4)	170 (8.7)	< 0.0001	402 (6.4)	328 (6.0)	0.33
Heart attack	445 (3.80)	225 (3.3)	125 (4.2)	95 (4.8)	0.001	226 (3.6)	219 (4.0)	0.27
Stroke	228 (1.95)	113 (1.7)	64 (2.2)	51 (2.6)	0.05	132 (2.1)	96 (1.8)	0.16
Arthritis	1813 (15.48)	856 (12.6)	527 (17.8)	430 (21.9)	< 0.0001	1058 (16.9)	755 (13.8)	< 0.0001
Infectious disease	1714 (14.63)	854 (12.6)	465 (15.7)	395 (20.1)	< 0.0001	932 (14.9)	782 (14.3)	0.35
**Multimorbidity, n (%)**	3496 (29.85)	1752 (25.8)	960 (32.4)	784 (40.0)	< 0.0001	1911 (30.6)	1585 (29.0)	0.06
**Falls, n (%)**	5291 (45.17)	2749 (40.5)	1428 (48.2)	1114 (56.8)	< 0.0001	2928 (46.9)	2363 (43.2)	< 0.0001
**Hearing problems, n (%)**	483 (4.12)	207 (3.0)	255 (8.6)	149 (7.6)	< 0.0001	308 (4.9)	175 (3.2)	< 0.0001
**Sight problems, n (%)**	875 (7.47)	327 (4.8)	127 (4.3)	293 (14.9)	< 0.0001	532 (8.5)	343 (6.3)	< 0.0001
**Limiting Pain, n (%)**	2172 (18.54)	886 (13.1)	660 (22.3)	626 (31.9)	< 0.0001	1242 (19.9)	930 (17.0)	< 0.0001
**Smoking, n (%)**								
Never	7095 (60.57)	4055 (59.7)	1822 (61.5)	1218 (62.1)	0.06	3827 (61.3)	3268 (59.8)	0.72
Former smoker	3231 (27.58)	1914 (28.2)	801 (27.0)	516 (26.3)		1651 (26.4)	1580 (28.9)	
Current smoker	1387 (11.84)	819 (12.1)	341 (11.5)	227 (11.6)		789 (12.3)	618 (11.3)	
**Alcohol, n (%)**								
Has never used	1118 (9.54)	589 (8.6)	315 (10.6)	220 (11.2)	< 0.0001	612 (9.8)	506 (9.3)	< 0.0001
Currently doesn’t use or mild user	8758 (74.77)	5024 (74.0)	2211 (74.6)	1523 (77.7)		4774 (76.4)	3984 (72.9)	
Moderate user	762 (6.51)	493 (7.3)	174 (5.9)	95 (4.8)		353 (5.7)	409 (72.9)	
Heavy user	1075 (9.18)	688 (10.1)	264 (8.9)	123 (6.3)		508 (8.1)	567 (10.4)	
**Unintentional weight loss, n (%)**	2339 (19.97)	1211 (17.8)	582 (19.6)	546 (27.8)	< 0.0001	1348 (21.6)	991 (18.1)	< 0.0001
**Hospitalization, n (%)**	1559 (13.31)	769 (11.3)	436 (14.7)	354 (18.1)	< 0.0001	867 (13.9)	692 (12.7)	0.05
**No hospitalization**	10,154 (86.7)	6019 (88.7)	2528 (85.3)	1607 (81.9)	< 0.0001	4774 (87.3)	5380 (86.1)	0.04
**1 to 5 hospitalizations**	1106 (9.4)	537 (7.9)	324 (10.9)	245 (12.5)		496 (9.1)	610 (9.8)	
**More than 5 hospitalizations**	453 (3.9)	232 (3.4)	112 (3.8)	109 (5.6)		196 (3.6)	257 (4.1)	
**Living alone, n (%)**	1154 (9.9)	422 (6.2)	414 (14.0)	318 (16.2)	< 0.0001	1067 (17.1)	87 (1.6)	< 0.0001

Data are presented as mean and standard deviation, or frequency and percentage

Quantitative comparisons were made by t-test or one-way Welch’s ANOVA. a: statistically significant difference compared to the without loneliness group by Dunnett’s t-test

Qualitative comparisons were made by Chi-square test

The incidence of all-cause mortality in participants without loneliness was 5.1% (95%CI: 4.6–5.6), in subjects with mild loneliness it was 6.1% (95%CI: 5.3–7.0), and in those with severe loneliness it was 9.2% (95%CI: 7.9–10.5) (*p* < 0.0001); and in the case of socially integrated subjects the incidence of all-cause mortality was 4.6% (95%CI: 4.0–5.1) and in subjects with social isolation it was 7.3% (95%CI: 6.7–7.9) (p < 0.0001). The incidence of all-cause mortality in subjects without loneliness and social integration was 4.1% (95%CI: 3.5–4.8), in participants without loneliness and social isolation was 6.1% (95%CI:5.3–6.9) in participants with mild loneliness and social isolation it was 7.4% (95%CI: 6.1–8.6) while in the participants with mild loneliness and social integrated it was 4.4% (95%CI:3.2–5.5), and in those with severe loneliness and social isolation it was 10.5% (95%CI: 8.8–12.2) while in the participants with severe loneliness and social integrated it was 7.0% (95%CI:5.1–8.8).

Correlation analyzes showed a significant correlation between loneliness and social isolation scores (gamma: 0.2, tau: 0.13, rho: 0.15; all *p* < 0.0001) indicating that when social isolation decreases, the feeling of loneliness decreases too. Loneliness and social isolation categories are weakly associated (Cramer’s V: 0.13, p < 0.0001), while living alone is moderately associated with loneliness (Cramer’s V: 0.15, p < 0.0001) and social isolation (Cramer’s V: 0.26, p < 0.0001).

Table 2 shows the results of the univariate logistic regression analyzes to determine the association of various sociodemographic, clinical, psychological, and lifestyle characteristics with all-cause mortality. All characteristics except financial status were associated in some way with all-cause mortality.

**Table 2:** Univariate regression analysis to determine the association with all-cause mortality of the Sociodemographic, clinical, psychological and lifestyle characteristics

	Regression coefficient	OR	95%CI	***p*** value
**Age**				
50–59	Reference			
60–69	0.94	2.55	1.86–3.48	< 0.0001
70–79	1.68	5.40	3.99–7.31	< 0.0001
80 and more	2.58	13.21	9.67–18.03	< 0.0001
**BMI, units**	−0.07	0.93	0.91–0.94	< 0.0001
**Sex (Women)**	−0.52	0.59	0.51–0.68	< 0.0001
**Schooling (ref. no schooling)**				
≥ 7 years	0.87	2.39	1.94–2.97	< 0.0001
1–6 years	1.19	3.30	2.58–4.22	< 0.0001
No schooling	Reference			
**Financial Situation**				
Excellent	Reference			
Very Good	−0.36	0.69	0.23–2.04	0.51
Good	−0.30	0.74	0.33–1.62	0.74
Fair	−0.08	0.91	0.42–1.98	0.82
Poor	0.45	1.57	0.71–3.46	0.25
**Cognition, score**	−0.80	0.44	0.40–0.49	< 0.0001
**≥1 activity of daily living affected**	1.89	6.63	5.36–8.20	< 0.0001
**Depressive symptoms**	0.61	1.85	1.58–2.16	< 0.0001
**Satisfaction with life (ref. agree)**				
Agree	Reference			
Neutral	0.30	1.35	1.05–1.74	0.02
Disagree	0.30	1.35	1.01–1.82	0.04
**Internal locus of control**	0.04	1.05	1.01–1.08	0.03
**Multimorbidity**	0.79	2.22	1.90–2.59	< 0.0001
**Infectious Diseases**	0.69	2.00	1.67–2.40	< 0.0001
**Falls**	0.41	1.51	1.29–1.76	< 0.0001
**Sight problems**	0.87	2.40	1.93–2.99	< 0.0001
**Hearing problems**	0.75	2.13	1.59–2.84	< 0.0001
**Limiting Pain**	0.44	1.55	1.30–1.85	< 0.0001
**Smoking**				
Never	Reference			
Former Smoker	0.37	1.45	1.23–1.71	< 0.0001
Current Smoker	0.07	1.07	0.84–1.38	0.55
**Alcohol consumption**				
Never used	Reference			
Currently doesn’t use or mild user	0.05	1.05	0.81–1.36	0.70
Moderate user	−0.31	0.73	0.48–1.11	0.14
Heavy user	−0.61	0.54	0.36–0.81	0.003
**Unintended weight loss**	0.65	1.90	1.61–2.25	< 0.0001
**Hospitalization**	1.05	2.86	2.41–3.40	< 0.0001
**Living alone**	0.32	1.38	1.10–1.73	0.006

Data presented as univariate analysis

OR: Odds Ratio, 95%CI: 95% confidence interval

Table 3 shows the results of the logistic regression models to determine the effect of loneliness and social isolation, as well as their interactions with all-cause mortality, it was observed in the unadjusted model that severe loneliness (OR = 1.73, 95% CI: 1.24–2.40, *p* = 0.001) and social isolation (OR = 1.50, 95%CI: 1.21–1.87, *p* < 0.0001) were associated with a higher probability of death, however, no interaction was observed between loneliness and social isolation that was associated with increased all-cause mortality. After multivariate adjustment for the sociodemographic, clinical, psychological and lifestyle characteristics associated with all-cause mortality, it was observed that only social isolation (OR = 1.30, 95%CI: 1.03–1.64, *p* = 0.03) was the factor associated with a higher probability of all-cause mortality, and the interaction between loneliness and social isolation continued to have no effect on all-cause mortality. The sensitivity shows in the table 4, similar results were found, showing that the effect size of social isolation is not due to data segregation into 3 loneliness categories.

**Table 3:** Logistic regression models to determine the effect of loneliness and social isolation on all-cause mortality

	Unadjusted model			Adjusted model^**a**^		
Loneliness	OR	95%CI	***p*** value	OR	95%CI	***p*** value
Without	Reference			Reference		
Mild	1.06	0.76–1.46	0.71	0.83	0.59–1.16	0.28
Severe	1.73	1.24–2.40	0.001	1.03	0.71–1.64	0.87
**Social Isolation**						
Integrated	Reference			Reference		
Isolated	1.50	1.21–1.87	< 0.0001	1.30	1.03–1.64	0.03
**Interactions between loneliness and social isolation**						
Without loneliness and Integrated	Reference			Reference		
Mild loneliness and isolated	1.15	0.77–1.70	0.48	1.14	0.76–1.73	0.53
Severe loneliness and isolated	1.04	0.69–1.55	0.84	0.99	0.64–1.51	0.94

a: Model adjusted by age, sex, schooling, more than one activity of daily living affected, depressive symptoms, satisfaction with life, internal locus of control, multimorbidity, infectious diseases, falls, sight problems, hearing problems, limiting pain, smoking, alcohol consumption, unintended weight loss, hospitalization and living alone

**Table 4:** Sensitivity analyses of the logistic regression models to determine the effect of the of any level of loneliness and social isolation on all-cause mortality

	Unadjusted model			Adjusted model^**a**^		
	OR	95%CI	***p*** value	OR	95%CI	***p*** value
**Loneliness**						
Without	Reference			Reference		
Mild or severe	1.31	1.01–1.69	0.04	0.92	0.69–1.21	0.54
**Social Isolation**						
Integrated	Reference			Reference		
Isolated	1.50	1.21–1.87	< 0.0001	1.31	1.03–1.65	0.03
**Interactions between loneliness and social isolation**						
Without loneliness and Integrated	Reference			Reference		
Loneliness and isolated	1.11	0.81–1.53	0.52	1.07	0.76–1.49	0.71

a: Model adjusted by age, sex, schooling, more than one activity of daily living affected, depressive symptoms, satisfaction with life, internal locus of control, multimorbidity, infectious diseases, falls, sight problems, hearing, limiting pain, smoking, alcohol consumption, unintended weight loss, hospitalization and living alone

## Discussion

In this retrospective observational study, we aimed to analyze the association of loneliness and isolation and their interaction with all-cause mortality in older adults, after multivariate adjustment for various covariates associated with all-cause mortality, we found that only social isolation was associated with an increased risk of all-cause mortality at a three-year follow-up, neither loneliness nor the interaction between loneliness and social isolation were associated with all-cause mortality.

Subjects with any degree of loneliness, as well as those with social isolation, presented higher proportions of alterations in mental health and physical health, other studies have also identified the risk of mental health problems such as depression, dementia, dissatisfaction with life, attempted suicide and abuse [49, 50], as well as greater alterations in physical health, since it has been found that participants with loneliness or isolation required more visits to the emergency room, hospital readmissions, risk of falls, and risk of malnutrition [4].

The cause of loneliness has been described in the theory of cognitive discrepancy, which indicates that loneliness is the cause of the mismatch between the actual quality and quantity of social interactions and those that the subject expects, this discrepancy can be caused by various social situations among them the same social isolation [51], this is the reason why loneliness and social isolation have been studied together, because they can be a synergistic or combined effect of isolation and loneliness, in our study we observed a relationship between both variables and we also have higher incidences of all-cause mortality when some level of loneliness was present in conjunction with social isolation, but when the risk of death is evaluated, through univariate logistic regression considering each variable together with its interaction, the association of loneliness and isolation with all-cause mortality was shown, but the interaction had no significant effect, the above is consistent with a lack of interdependence between these two aspects of socialization and support the idea that each one (loneliness and social isolation) has their own independent pathway to all-cause mortality. After multivariate adjustment, neither interaction nor loneliness had a significant effect on all-cause mortality, only social isolation was associated with increased mortality odds, these findings speak to those where the combined effect is not worse than experiencing either by itself [28, 29] and those of Gilmore and Ramage [52] who did not find a significant interaction between isolation and loneliness; and recently Ward et al. in 2021 showed that subjects with social isolation but not loneliness (HR = 1.37, 95%CI: 1.04–1.81) had the same mortality risk as subjects with loneliness and social isolation present at the same time (HR = 1.43, 95%CI:1.09–1.87) [5]; in the same line of thought, Stokes et al. in 2021, observed in a 10-year follow-up that only social isolation (HR = 1.13, 95%CI: 1.04–1.23) but not loneliness (HR = 1.05, 95%CI: 0.93–1.18) had a significant association with all-cause mortality [29]. Therefore, although social isolation and loneliness can coexist, and there is evidence showing that they may be correlated [53], it can be suggested that social isolation may be the cause of the increased risk of all-cause mortality.

The reasoning for which it has been considered that loneliness can predict mortality has been mainly mentioned the risk of physical inactivity, depression and defective immune functioning [54, 55] which are associated with worse outcomes or the development of diseases in older adults, on the other hand, a decrease in social interaction can affect the search for appropriate medical treatment, establish non-adherence to medications and, thus, developing unhealthy behaviors [56]. Also, socially isolated, or lonely older adults, have an increased perception of threats and vulnerability, this hypervigilance alters the psychological self-regulatory processes that influence physiologic functions, undermining sleep quality; the aforementioned, combined with unhealthy behaviors observed in socially isolated elderly such as smoking, drinking, obesity and physical inactivity [5], would increase the risk of morbidity and mortality [5, 26]. Studies showing a greater affinity of isolation with mortality than loneliness suggest that behaviors related to health care such as less use or access to medical care may be the cause of increased mortality [5], consistent with this, in our study we observed fewer hospitalizations of isolated participants than those with severe loneliness, which could suggest a relationship between this lack of medical care and the results we observed.

There are important cultural and socioeconomic moderators of the relationship between social isolation and loneliness, and health and mortality. On the one hand, evidence suggests cultural individualism or collectivism moderates the effect of loneliness on health and mortality. Individualism refers to valuing and striving for autonomy and placing one’s personal goals above those of others. Collectivism refers to seeing the interests of one’s group, such as one’s family or community, as more important than those of oneself [57]. Studies have found the effect of loneliness on health could be stronger in more collectivistic countries [58] like Latin American countries, current evidence shows that in Latin America loneliness has a small significant effect on mortality (HR = 1.13, 95%CI:1.01–1.26, I^2^ = 10.1%) [10], but without considering social isolation, that is why research in Latin America is urgent in order to have a complete vision of the effects of loneliness and social isolation on all-cause mortality.

In Mexico, the study of loneliness in older adults is relatively recent, the main findings are its association with chronic-degenerative diseases such as renal failure, diabetes, and hypertension [13], and regarding social isolation it was found, in a recent study, an association with all-cause mortality after a 12-year follow-up [59].

Our findings emphasize how important it is for social and health-care policy makers to develop effective intervention programs to reduce social isolation among older adults, especially considering the current context of the COVID-19 pandemic. Thus, it is critical to increase awareness about the impact that social isolation has on health to design interventions that can help older adults regain or maintain social activities and to develop strategies to remain socially connected [60].

The main limitation of this study was the lack of all-cause mortality follow-up dates and dates of the beginig of the exposition, which limited the performance of a Cox proportional hazards model, other limitations are the self-report of clinical characteristics such as diagnoses of comorbidities, activities of the daily life affected, history of fall, weight loss and previous hospitalizations, and there was also no medical information available at the time of the survey. In addition, the MHAS is a representative sample, but it is only representative of the Mexican population, therefore, more studies must be carried out in Latin American countries with which the findings of this study can be contrasted.

The strengths of this study is the large representative study sample from Mexico that allows a better estimation of the effect sizes controlling for various covariates, likewise, this study is one of the first and largest in a highly collectivistic as well middle-low-income country, implying that our findings may help resolve conflicts about the effect of loneliness and social isolation globally.

## Conclusion

Social isolation, but not loneliness or their interaction, was associated with all-cause mortality in Mexican adults older than 50 years. This finding may help direct possible future interventions that help improve mental health in older adults from a highly collectivistic country.

## Data Availability

The datasets analyzed for this study are of public use and can be found in the Mexican Health an Aging Study (MHAS) website http://www.mhasweb.org/.
